# The Roles of Early Response and Sudden Gains on Depression Outcomes: Findings From a Randomized Controlled Trial of Behavioral Activation in Goa, India

**DOI:** 10.1177/2167702619825860

**Published:** 2019-02-15

**Authors:** Daisy R. Singla, Steven D. Hollon, Christopher G. Fairburn, Sona Dimidjian, Vikram Patel

**Affiliations:** 1 Department of Psychiatry, University of Toronto, and Sinai Health System, Toronto, Canada; 2 Department of Psychology, Vanderbilt University; 3 Department of Psychiatry, Warneford Hospital, University of Oxford; 4 Department of Psychology and Neuroscience, University of Colorado Boulder; 5 Sangath Center, Goa, India; 6 Department of Global Health and Social Medicine, Harvard Medical School; 7 Department of Global Health and Population, Harvard TH Chan School of Public Health

**Keywords:** early response, sudden gains, behavioral activation, psychological treatment, depression, nonspecialist provider, low- and middle-income country

## Abstract

The Healthy Activity Program (HAP), a brief, lay-counselor-delivered, behavioral activation psychological treatment, was found to be effective in reducing depressive symptoms among primary care attendees in India. We now examine whether early response predicts depression (PHQ-9) outcomes at the primary endpoint of 3 months and sustained recovery at 12 months after enrollment and the extent to which this effect is influenced by sudden gains in the context of the larger randomized controlled trial. HAP participants (*N* = 245) who exhibited an early response (150 of 245 or 61.2%), as defined by a 50% reduction in depressive symptoms from baseline to Session 3, had lower depressive symptom scores than those who did not at 3 months (5.29 vs. 10.75, *F* = 33.21, *p* < .001) and at 12 months (6.56 vs. 11.02, *F* = 21.84, *p* < .001). Further exploratory analyses suggested that this advantage was largely confined to the subset of early responders who also showed sudden gains (87 of 150).

Depression is the leading cause of disability among adults worldwide (Ferrari et al., 2013). Psychological treatments delivered by nonspecialist providers have been successful at reducing the burden of adult depression both in low- and middle-income countries (LMICs; Singla et al., 2017) and in high-income countries (Hoeft, Fortney, Patel, & Unützer, 2016), and they are recommended as a first line of treatment (World Health Organization, 2015). It is not clear, however, how these treatments achieve their effects. Identifying variables that predict depression outcome may help clinicians anticipate individual patient trajectories and guide the development of more effective interventions (Kazdin, 2009; Kraemer, 2016). Potential variables of interest include the related but distinct concepts of early response and sudden gains, which have each been shown to predict improved acute and sustained treatment outcomes for depression (e.g., Aderka, Nickerson, Bøe, & Hofmann, 2012; Crits-Christoph et al., 2001; Fairburn, Agras, Walsh, Wilson, & Stice, 2004; Grilo, White, Wilson, Gueorguieva, & Masheb, 2012; Lemmens, DeRubeis, Arntz, Peeters, & Huibers, 2016; Lutz et al., 2014; Lutz, Stulz, & Köck, 2009). However, the two constructs have rarely been examined in the same study and have never been examined with respect to brief, psychological treatments delivered by nonspecialist providers.

The current study explored the role of early response and sudden gains within a randomized controlled trial (RCT) evaluating the Healthy Activity Program (HAP), a brief, lay-counselor-delivered, behavioral activation (BA) treatment for patients with depression in primary care (Chowdhary et al., 2015). This trial was conducted in Goa, India. The trial found that among primary care attendees with moderately severe to severe depressive symptoms, HAP was more effective than enhanced usual care in reducing depressive symptoms and improving remission rates (Patient Health Questionnaire–9 [PHQ-9] < 10) at the primary endpoint of 3 months (Patel et al., 2017) and at 12 months after enrollment (Weobong et al., 2017).

## Early response as a predictor for clinical outcomes

Over the past two decades, psychotherapy researchers have examined early treatment effects and early patterns of change. Multiple studies across varying disorders and treatment modalities have revealed that a substantial portion of participants experience response early in treatment, and this early response is associated with a positive outcome at treatment termination that is largely sustained over time (e.g., Aderka et al., 2012; Crits-Christoph et al., 2001; Grilo et al., 2012; Lutz et al., 20089, 2014). Several RCTs in both naturalistic and controlled environments have demonstrated similar results for depressive disorders specifically and across varying measures. For example, Lutz et al. (2009) reported that moderate to severely depressed patients with a rapid positive change showed reliable improvement on the Beck Depression Inventory (BDI; Beck, Ward, Mendelson, Mock, & Erbaugh, 1961) at treatment termination and maintained their initial improvement over an 18-month follow-up period. Likewise, Tadić et al. (2010) demonstrated that early response was a highly sensitive predictor of later stable response and remission on the Hamilton Rating Scale for Depression (Hamilton, 1960), among patients with major, minor, or subsyndromal depression undergoing cognitive behavioral therapy (CBT). Despite the influence of early response on clinical outcomes and a growing interest in routine outcome monitoring (e.g., Clark et al., 2018; Lutz, De Jong, & Rubel, 2015), there is no widely recognized definition of early response in psychotherapy research and there are considerable differences in the definitions used. For example, Renaud and colleagues (1998) defined early response as a decline of ≥ 50% on the BDI score from baseline until the second session of psychotherapy, whereas others defined early response in terms of a reduction of symptoms by the third or fourth session (Fennell & Teasdale, 1987; Grilo et al., 2012).

## Sudden gains as a predictor for clinical outcomes

The separate but somewhat related concept of sudden gains also has been extensively studied in the psychological treatment literature. Tang and DeRubeis (1999) were the first to define sudden gains, demonstrating that a subset of patients who responded to CBT showed a sudden drop in depression scores between two consecutive treatment sessions rather than responding gradually across sessions. These sudden gains, based on absolute, relative, and stable between-session symptom improvement, predicted not only better treatment outcome at termination but also greater stability of response among patients who showed comparable overall response in a more continuous fashion (Tang et al., 2007). Similar to early response, there is accumulating evidence to suggest that sudden gains represent a common phenomenon among depression and anxiety patients that influence clinical outcome across treatment modalities (Aderka et al., 2012; Lemmens et al., 2016) including BA-based psychological treatments for depression (Masterson et al., 2014). Sudden gains can overlap with early response because a substantial proportion of abrupt positive shifts have been found to occur in the early phase of psychotherapy (e.g., Busch, Kanter, Landes, & Kohlenberg, 2006; Kelly, Roberts, & Ciesla, 2003; Lutz et al., 2013). For example, Busch and colleagues (2006) found that reductions in depressive symptoms posttreatment were predicted by first-session gains (gains that occurred between the first and second sessions of treatment) and sudden gains occurring in the first half of treatment. Similar to findings in the early response literature, this finding highlights the importance of early change. Despite the potential overlap between early response and sudden gains, and the growing evidence supporting each, few studies have compared these two concepts within a single sample or examined sudden gains within the context of individuals who have met early response. Furthermore, reliable predictors of early response and sudden gains have not been identified (Grilo, Masheb, & Wilson, 2006; Lemmens et al., 2016). Finally, no study to our knowledge has examined the roles of early response or sudden gains within the context of a nonspecialist-delivered psychological treatment.

In the current study, we examined the predictive utility of early response among patients with moderate to severe depressive symptoms and its relation to sudden gains among primary care patients receiving HAP by nonspecialist lay counselors. Our primary questions included the following:

1. Does early response occur in HAP and, if so, in what proportion of patients?
2. What patient and treatment variables are related to early response?
3. Does early response predict symptom scores at 3 and 12 months postenrollment?
4. What is the relationship between early response and sudden gains in this sample?

We hypothesized that early response would be seen among HAP participants at a similar rate as in other psychological treatment studies (40%–50%; Fairburn et al., 2004; Grilo et al., 2012). We also hypothesized that baseline severity and patient education level would be significantly related to early response (i.e., individuals who had higher levels of education and lower levels of baseline severity would be quicker to grasp concepts related to the HAP treatment). In addition, we hypothesized that individuals who met our definition of early response and sudden gains criteria would have lower depressive symptom scores at both 3 and 12 months after enrollment. Finally, we expected some overlap between those who met our criteria for early response and sudden gains.

## Methods

### Setting

This study was embedded in the HAP trial, which was conducted in 10 primary health centers in the state of Goa, India. HAP was part of PREMIUM (PRogram for Effective Mental health Interventions in Under-resourced health systeMs)—a series of studies that aimed to develop and evaluate brief, contextually appropriate psychological treatments suitable for delivery by lay counselors in routine care (Patel et al., 2010). HAP patients were 18 to 65 years old and had moderately severe to severe depressive symptoms on the PHQ-9 (Spitzer, Kroenke, & Williams, 1999), which has been previously validated in this context (Patel et al., 2008). Selection procedures including inclusion and exclusion criteria, patient characteristics, and outcomes are detailed elsewhere (Patel et al., 2010, 2017). The HAP manual and training website can be accessed online (premium.nextgenu.org). Institutional Review Boards at Sangath, the London School of Hygiene and Tropical Medicine, and the Indian Council of Medical Research provided ethical approval.

### Procedures

#### Samples

The present study involved all patients who were randomized to the HAP treatment arm (*N* = 245). This is similar to other studies that have examined early response within one treatment arm within a larger RCT (e.g., Kelly, Cyranowski, & Frank, 2007).

#### Treatment

HAP is a behavioral activation psychological treatment (Dimidjian, Barrera, Martell, Muñoz, & Lewinsohn, 2011) culturally adapted for the local context (Chowdhary et al., 2015). HAP is a six- to eight-session treatment, in which an additional two sessions (up to a maximum of eight) could be added during the middle phase of treatment (Sessions 3 to 5) if patients were not making adequate clinical progress. Progress was assessed by the lay counselors on the basis of the patients’ session-wise PHQ-9 scores (PHQ-9 > 10 over two consecutive sessions; *n* = 59 received seven or eight sessions; Chowdhary et al., 2015). HAP was delivered in three phases (beginning, middle, and end). Key strategies included psychoeducation, behavioral assessment, activity monitoring, activity structuring and scheduling, activation of social networks, and problem solving (Chowdhary et al., 2015; Patel et al., 2017).

Lay counselors with no previous experience in mental health care were trained to deliver HAP and conduct peer supervision. They included members of the local community who were older than 18 years of age and had completed at least a high school education. The counselors underwent a 3-week participatory workshop covering the HAP program, followed by an internship phase of 6 months, in which trainee counselors delivered the treatment to eligible patients in primary health centers. Eleven counselors who met competency standards participated in the trial. They were subject to peer-led supervision in groups of four to five counselors as well as bimonthly individual supervision with an expert supervisor. Supervision involved rating peers’ audio-recorded individual sessions for therapy quality. Additional details regarding the training and supervision procedures of lay counselors can be found elsewhere (Singla et al., 2014).

#### Dependent variable

The primary outcome was depressive symptoms on the PHQ-9 (Spitzer et al., 1999) at 3 months and 12 months after enrolment as assessed by independent evaluators blind to treatment status. The PHQ-9 is usually administered via self-report, but because many participants were illiterate, the instrument was administered verbally. In addition, the lay counselors assessed PHQ-9 session-wise scores at the beginning of each treatment session.

#### Independent variables

##### Early response

The definition of early response was informed by previous studies and defined as a 50% reduction in depressive symptom severity by the third session (Grilo et al., 2012). We selected the third session a priori because individuals would have attended at least two HAP sessions and entered the middle phase of treatment. We later conducted receiver-operating characteristic (ROC) curves to determine whether this a priori definition was statistically supported (see the Analyses section below).

##### Sudden gains

Sudden gains were defined as an absolute decrease of 5 or more points on the PHQ-9 between two consecutive sessions (e.g., Session 1 and 2), a relative decrease of 25% between session-wise scores between the same or following sequential sessions (e.g., Session 2 and 3), and a stable decrease in the participant’s session-wise mean depression scores (i.e., no subsequent session could have a higher PHQ-9 score than a previous one). The first and second criteria have been used in another BA trial that used the PHQ-9 to assess depressive symptoms (Masterson et al., 2014). We modified the third criterion—originally defined as a significant difference between the average depressive symptom scores of 3 preceding weeks and the 3 following weeks as assessed by an independent-samples *t* test (Tang & DeRubeis, 1999)—to offer a more pragmatic assessment that could be used in a wider public health context, including those implemented by nonspecialist providers. Baseline characteristics related to the patient (age, education, marital status, occupation, and PHQ score) and treatment (average session duration and treatment dosage) were examined as potential covariates.

#### Data collection

Independent interviewers assessed depression outcomes at baseline and at the 3- and 12-month endpoints. All were blind to treatment arm status. Lay counselors collected the in-session PHQ-9 scores on a session-by-session basis. Both groups collected data using computer tablets that were uploaded in real time to a server reviewed for quality assurance by independent data managers. All patients provided informed consent.

### Analyses

All analyses were exploratory, using SAS 9.4. Missing data imputation procedures were used in SAS 9.4 (PROC MI and PROC MI ANALYZE) among those participants who were lost to follow-up at the primary endpoint of 3 and 12 months after study enrollment (*n* = 15 in HAP).

#### Defining early response

**Fig. 1.**
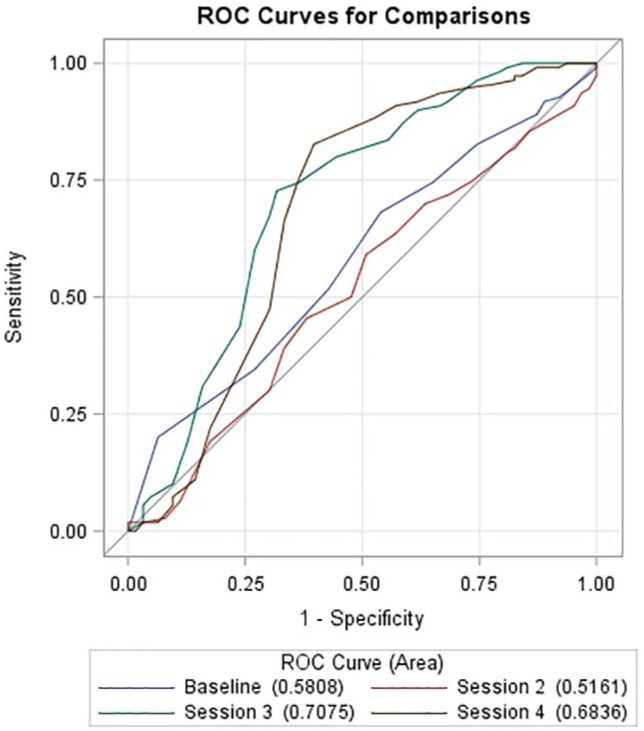
Receiver-operating characteristic (ROC) curves predicting depression remission (Patient Health Questionnaire–9 [PHQ] < 10) at posttreatment (determined by session-wise PHQ-9 scores) observed at baseline and Session 4. ROC curve for Session 3 is the most predictive (shown in green) with an area under the curve of 0.708 (*SE* = 0.044), with a 95% confidence interval of [0.621, 0.794].

Our definition of early response was supported by calculating ROC curves using reduced depressive symptom severity through each of the first four sessions (determined using absolute session-wise PHQ-9 scores) to predict the primary posttreatment outcome of remission (PHQ-9 < 10) from depression, as had been done in previous studies (Fairburn et al., 2004; Grilo et al., 2012). ROC curves can be used to test the accuracy of different measures (in this case, percentage reductions in depressive symptom severity) used to predict posttreatment outcome of interest. Figure 1 shows the ROC curves. As expected, ROC curves at Session 3 emerged as the most predictive on the basis of the overall area under the curve (AUC; Streiner & Cairney, 2007). We found that an AUC = 0.708 (*SE* = 0.044, 95% confidence interval [CI] = [0.621, 0.794]) indicated moderate accuracy (Streiner & Cairney, 2007). Inspection of this ROC revealed that a reduction of ≥ 50% in PHQ-9 depressive symptom severity by the third session maximized sensitivity and 1 – specificity (0.865 and 0.600, respectively).

#### Comparison of participants with and without early response

*t* tests and chi-square tests were used to test for differences between participants who showed early response versus those who did not. Pearson correlations were used to assess the associations among the independent variables. If significant associations were observed (*p* < .05), linear mixed models (described next) adjusted for these variables.

#### Early response prediction of depression outcomes

We used generalized linear mixed models to estimate the effects of early response status on depression outcomes at 3 months and 12 months while controlling for baseline PHQ-9 scores and treatment dosage. We also used least square means to calculate standardized mean differences and effect sizes of depression outcomes between those who did, versus those who did not, show early response. We conducted the same set of analyses among those who met criteria for sudden gains, compared with those who did not.

#### Early response and sudden gains

We compared the frequencies of participants who met early response and sudden gains criteria, neither, or one or the other. Finally, Pearson correlation was used to assess the statistical association between early response and sudden gains status.

## Results

### Comparison of participants with and without early response

Of the patients, 61.22% (*n* = 150 of 245) met the criteria for early response. Their characteristics are detailed in Table 1. Participants who exhibited early response were aged 43.15 years (95% CI = [41.24, 45.12]; range = 18–65 years) and mostly female (76.67%), married (67.33%), and Hindu (93.33%). Their baseline depressive symptoms were an average of 18.21 on the PHQ-9, and their average treatment dosage was 5.53 sessions (95% CI = [5.22, 5.83]). Participants who met criteria for early response attended significantly more sessions than those who did not (5.53, 95% CI = [5.31, 5.82] vs. 3.76 sessions, 95% CI = [3.11, 4.44], *t* = −5.18, *p* < .001). No other differences were observed between those who met criteria for early response and those who did not, including baseline PHQ-9 scores. The same relation was found for those with sudden gains, which also was significantly related to treatment dosage (*r* = .333, *p* < .001) but no other patient or treatment characteristic.

**Table 1.** Patient and Treatment Variables and Depression Outcomes of Early Responders Versus Non–Early Responders and Sudden Gainers Versus Non–Sudden Gainers (*N* = 245)

Variable	Early responders (*n* = 150)	Non–early responders (*n* = 95)
Patient		
Age	43.15 [41.24, 45.12]	41.27 [38.71, 43.85]
Female gender (%)	76.67	76.84
Marital status: married (%)	67.33	68.42
Religion: Hindu (%)	93.33	89.47
Treatment		
Baseline depressive symptoms	18.21 [17.81, 18.73]	17.52 [17.14, 18.05]
Average dosage (number of sessions)^a^	5.53 [5.24, 5.81]	3.76 [3.12, 4.47]
Average session duration (min)	38.77 [37.70, 39.91]	38.1 [34.91, 41.43]

Values are means with 95% confidence intervals in brackets unless otherwise specified.

a Significant difference between early gainers and non–early gainers (*t* = −5.18, *p* < .001).

### Early response prediction of depression outcomes

HAP participants who met criteria for early response (*n* = 150) had significantly lower depressive symptom scores at 3 months (PHQ-9 = 5.89, *SD* = 6.11 vs. 10.75, *SD* = 7.95, *p* < .001) and sustained outcomes at 12 months (PHQ-9 = 6.56, *SD* = 6.38 vs. 11.02, *SD* = 7.08, *p* < .001) than those who did not. In the linear regression analyses (see Table 2), we also found that early response status predicted reduced depression at 3 months, even after controlling for baseline PHQ-9 scores and treatment dosage (*F* = 33.21, *p* < .0001). We found similar results when examining the effect of early response on 12-month depression outcomes (*F* = 21.84, *p* < .0001).

**Table 2.** Effects of Early Response Status on Depression Outcomes (*N* = 245)

Variable	Early responders (*n* = 150)	Non–early responders (*n* = 95)	Point estimate^b^	*p* value	Effect size
Mean depressive symptoms at 3 months	5.29 (0.566)	10.75 (0.723)	*F* = 33.21 *SMD* = −5.46	< .0001	.781
Mean depressive symptoms at 12 months	6.56 (0.552)	11.02 (0.740)	*F* = 21.84 *SMD* = −4.46	< .0001	.638

Depressive symptoms were measured using the Patient Health Questionnaire–9. Values in parentheses are standard errors. SMD = standardized mean difference.

a *F*-value of early gains status predicting outcome.

#### Early response and sudden gains

**Fig. 2.**
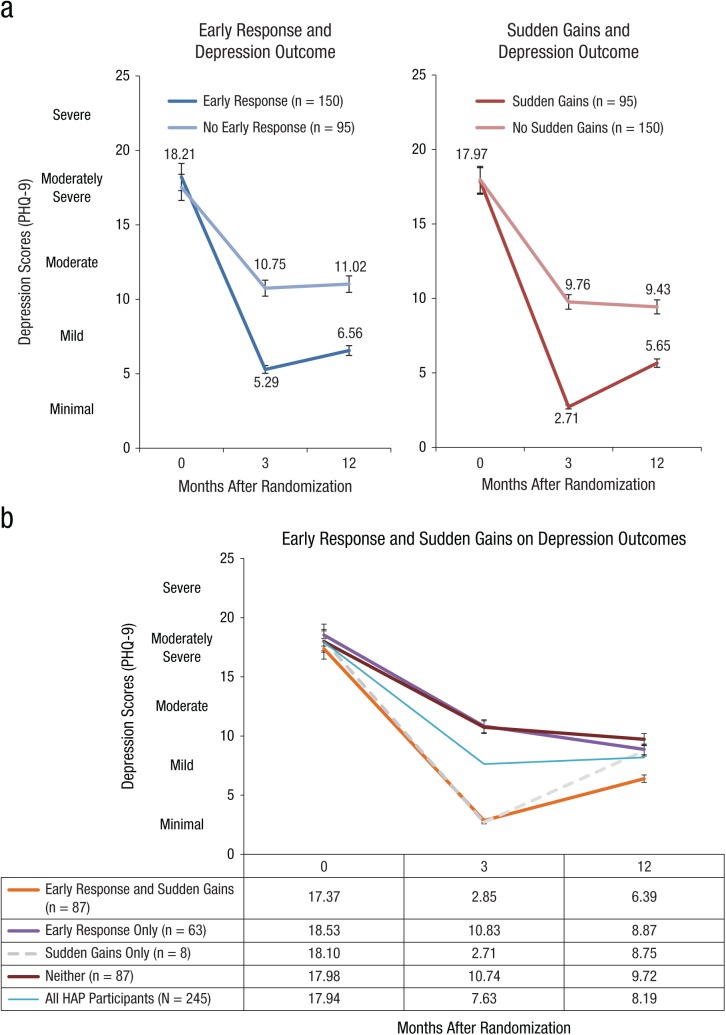
(a) Early responders versus non–early responders and sudden gains versus non–sudden gains on depression outcomes. (b) The relation between early response and sudden gains on depression outcomes. HAP = Healthy Activity Program.

As shown in Figure 2a, sudden gains were largely subsumed within early response but not the other way around. Although only slightly more than half of the patients who met criteria for early response also met criteria for sudden gains (87 of 150, or 58.0%), they largely accounted for the predictive utility of early response and demonstrated the lowest depression scores at both remission and recovery at 3 and 12 months, respectively (Fig. 2b). Participants who showed early response in the absence of sudden gains (*n* = 63) differed little from participants who showed neither early response nor sudden gains (also *n* = 87) in terms of depression scores at either 3 or 12 months. The few participants who did not meet criteria for early response but did subsequently show sudden gains (*n* = 8) were doing well at month 3 but did not sustain those gains through month 12.

## Discussion

Our objective was to determine whether early response predicted subsequent depression outcomes among primary care adult attenders with moderately severe to severe depression who received HAP as delivered by nonspecialist lay counselors in Goa, India. To do this, we examined the frequency of early response (defined as a 50% reduction in PHQ depressive symptom severity by the third treatment session) and its predictive role on the severity of depression at the primary endpoint of 3 months and sustained depression outcomes at 12 months after enrollment. We also explored the influence of sudden gains (defined as absolute, relative, and stable decreases in session-wise depressive symptom scores) among those patients who exhibited early response. Our findings showed that early response (*n* = 150 or 61.2% of all HAP participants) did indeed predict subsequent treatment outcome at both 3 and 12 months after enrollment but that this was largely due to those early responders who also showed sudden gains (*n* = 87 or 58.0% of all early responders; 35.5% of all patients receiving HAP). Thus, although there appeared to be overlap between those who met early response and sudden gains criteria, a closer examination demonstrated that those who met criteria for sudden gains largely accounted for the relationship between early response and treatment outcome at 3 and 12 months, respectively.

The proportion of early responders in our sample (61.2% of HAP participants) was similar to that in other studies using similar criteria (e.g., Grilo et al., 2012). Early response predicted lower depression scores at 3 and 12 months, even when controlling for treatment dose. Similar to previous psychotherapy trials for depression (Kelly et al., 2007; Tang & DeRubeis, 1999; Wilson, Fairburn, Agras, Walsh, & Kraemer, 2002), these results speak to the importance of both early change and treatment dosage as both independently predicting reduced depressive symptoms posttreatment. Given the way that we defined early response (a 50% reduction in symptom scores by Session 3), it is likely that the relation to treatment dose was an artifact of attrition: patients who dropped out before Session 3 could not meet criteria for early response regardless of how well they were doing up until that point (although, in point of fact, none of the dropouts were doing all that well). Another way to interpret this finding was that individuals who met criteria for early response or sudden gains were more likely to stay in treatment. As in other psychotherapy trials (e.g., Grilo et al., 2006; Kelly et al., 2007; Lemmens et al., 2016), we found that early response was not associated with specific patient or treatment characteristics such as baseline depression scores, marital status, patient gender, or the lay counselor delivering the therapy. Thus, apart from treatment dosage, it remains unclear which HAP participants are likely to exhibit either early response or sudden gains.

There was largely a one-sided relation in the overlap between early response and sudden gains; whereas only slightly more than half of HAP participants who met criteria for early response also showed sudden gains (87 of 150 or 58.00%); however, virtually all patients who showed sudden gains also met criteria for early response (87 of 95 or 91.58%), and it was these latter patients who also showed sudden gains who largely accounted for the predictive capacity of early response. Specifically, although early response (*n* = 150) by the third session predicted acute and sustained responses at 3 and 12 months, respectively, this effect was largely accounted for in the 58% (*n* = 87) of participants who also showed sudden gains. Furthermore, virtually all participants who showed sudden gains showed early response, but this was not the case when considering the number of individuals with early response who showed sudden gains (87/150). The mechanisms underlying this phenomenon merit further exploration in future research that might focus on potential candidates such as therapeutic alliance (Wucherpfennig, Rubel, Hofmann, & Lutz, 2017); coping skills (including increased patient activation levels: Weobong et al., 2017); reduced rumination (Shahar, Britton, Sbarra, Figueredo, & Bootzin, 2010), or enhanced social support (Singla, Kumbakumba, & Aboud, 2015). That being said, the practical implications of our findings are clear: Whereas early response can predict subsequent outcomes, sudden gain leading to early response is a more powerful predictor still. Further, early response is easy to compute, whereas sudden gains requires a somewhat more complicated calculation, but it is still within the capacity of most lay counselors in LMIC settings, at least in the simplified version that we computed.

We must acknowledge several limitations. First, we cannot assess the potential influences of early response or sudden gains over a longer treatment course because HAP was limited to a maximum of eight sessions. This treatment dose was deemed appropriate for the current study context (Chowdhary et al., 2015); is similar to other, effective nonspecialist-delivered psychological treatments for depression in LMICs (Singla et al., 2017); and led to clinical outcomes comparable with those observed in BA trials with at least twice as many sessions (Dimidjian et al., 2006; Richards et al., 2016). Second, there may be other correlates that we did not assess in the current study that could influence early response. For example, we did not assess the role of therapeutic alliance, which has been found to have a moderate association with early response (Wucherpfennig et al., 2017). Finally, because many participants in the current study context were illiterate, depressive symptoms were estimated using the PHQ-9 read aloud to each participant.

In summary, this study found that early response to a brief, psychological treatment for depression is a predictor of subsequent response. It is novel because this research was conducted in the context of a large RCT assessing one lay-counselor-delivered treatment, in an LMIC primary care setting and replicated similar observations of longer psychological treatments delivered by mental health professionals in high-income country specialist care settings. Much of this effect is due to the sudden gains occurring early in treatment, demonstrating the incremental validity of calculating sudden gains among those who meet early response. Stepped-care approaches that step up patients who do not show such early response to an alternative or more specialized treatment are a potential pragmatic implication of these findings. Further research is needed to better understand the mechanisms of these effects and how early response and especially sudden gains may be enhanced.
